# Assessment of tumor heterogeneity: an emerging imaging tool for clinical practice?

**DOI:** 10.1007/s13244-012-0196-6

**Published:** 2012-10-24

**Authors:** Fergus Davnall, Connie S. P. Yip, Gunnar Ljungqvist, Mariyah Selmi, Francesca Ng, Bal Sanghera, Balaji Ganeshan, Kenneth A. Miles, Gary J. Cook, Vicky Goh

**Affiliations:** 1 Division of Imaging Sciences and Biomedical Engineering, King’s College London, London, UK; 2 Department on Oncology, Guy’s & St Thomas’ NHS Foundation Trust, London, UK; 3 Paul Strickland Scanner Centre, Mount Vernon Hospital, London, Middlesex UK; 4 Clinical Imaging Sciences Centre, Brighton and Sussex Medical School, Brighton, Sussex, Falmer UK; 5 Division of Imaging Sciences & Biomedical Engineering, King’s College London, PET Imaging Centre, London, UK; 6 Chair of Clinical Cancer Imaging, Lambeth Wing, St Thomas Hospital, Lambeth Palace Road, London, SE1 7EH UK

**Keywords:** Texture analysis, Fractal analysis, Cancer, CT, MRI, PET

## Abstract

**Background:**

Tumor spatial heterogeneity is an important prognostic factor, which may be reflected in medical images

**Methods:**

Image texture analysis is an approach of quantifying heterogeneity that may not be appreciated by the naked eye. Different methods can be applied including statistical-, model-, and transform-based methods.

**Results:**

Early evidence suggests that texture analysis has the potential to augment diagnosis and characterization as well as improve tumor staging and therapy response assessment in oncological practice.

**Conclusion:**

This review provides an overview of the application of texture analysis with different imaging modalities, CT, MRI, and PET, to date and describes the technical challenges that have limited its widespread clinical implementation so far. With further efforts to refine its application, image texture analysis has the potential to develop into a valuable clinical tool for oncologic imaging.

***Teaching Points*:**

• *Tumor spatial heterogeneity is an important prognostic factor.*

• *Image texture analysis is an approach of quantifying heterogeneity.*

• *Different methods can be applied, including statistical-, model-, and transform-based methods.*

• *Texture analysis could improve the diagnosis, tumor staging, and therapy response assessment.*

## Introduction

### Tumor heterogeneity

Imaging is used widely in oncologic practice for lesion characterization, confirmation of diagnosis, staging, treatment planning, targeting therapy, assessing treatment response, and surveillance. Diagnosis and staging are typically based on a lesion’s anatomical appearance and the extent of tumor spread on imaging. Different imaging modalities, such as X-ray, ultrasound (US), computed tomography (CT), magnetic resonance imaging (MRI), and positron emission tomography (PET), can be used singly or in combination, depending on the tumor type, site, and clinical question to be answered. A limitation that applies to all imaging modalities is that image intepretation is based on a visual process. Yet, there are features within each image that may not be appreciated readily by the naked eye. Furthermore, when images are analyzed in a more quantitative manner, standard region of interest analysis may provide a mean parameter value, e.g., Hounsfield unit (HU) on CT, signal intensity (SI) on MRI, or standardized uptake value (SUV) on PET, but does not typically describe the underlying spatial distribution.

**Fig. 1:**
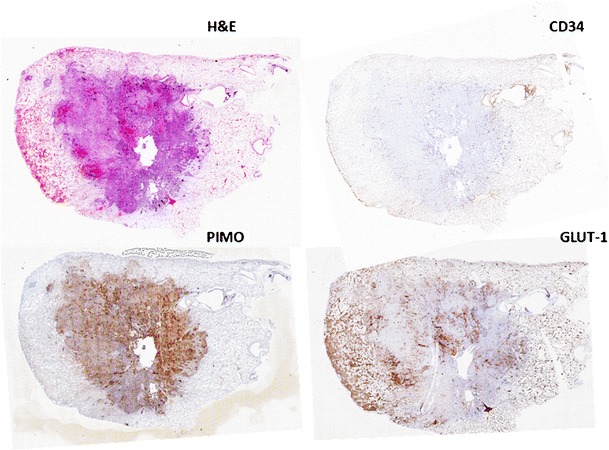
Non-small-cell lung cancer showing spatial variation in staining for angiogenesis (CD34), pimonidazole (hypoxia), and glucose transporter protein expression (Glut-1)

Tumors are heterogeneous both on genetic and histopathological levels (Fig. 1) with intratumoral spatial variation in the cellularity, angiogenesis, extravascular extracellular matrix, and areas of necrosis. Tumors with high intratumoral heterogeneity have been shown to have poorer prognosis, which could be secondary to intrinsic aggressive biology or treatment resistance [1–3]. It is difficult to assess intratumoral heterogeneity with random sampling or biopsy as this does not represent the full extent of phenotypic or genetic variation within a tumor. Thus, a non-invasive method of assessing the heterogeneity within a tumor might be of clinical benefit, particularly in this age of personalized medicine, to select poor prognosis patients for more intensive therapy. Hence, tumor heterogeneity is a clinically relevant parameter for imaging that may be quantifiable and that could augment standard reporting methods.

### Texture analysis

Texture analysis refers to a variety of mathematical methods that can be used to evaluate the gray-level intensity and position of the pixels within an image to derive so-called ‘texture features’ that provide a measure of intralesional heterogeneity [4]. Different methods have been applied, including statistical-, model-, and transform-based methods [5–12]. Statistical-based techniques have been most commonly applied and describe the distribution and relationships of gray-level values in the image. Three orders of parameters are described in statistical-based texture analysis. First-order statistics relate to gray-level frequency distribution within the region of interest, which can be obtained from the histogram of pixel intensities [13]. It is dependent on a single pixel value rather than its interaction with neighboring pixels. First-order statistics, based on histogram analysis, include mean intensity, maximum intensity, minimum intensity, uniformity (uniformity of gray-level distribution), entropy (irregularity of gray-level distribution), standard deviation of the gray-level histogram distribution, skewness (asymmetry of the histogram), and kurtosis (flatness of the histogram) (Appendix). Second-order statistics are co-occurrence measurements calculated using spatial gray-level dependence matrices. These matrices determine how often a pixel of intensity *i* finds itself within a certain relationship to another pixel of intensity *j*. Second-order statistics based on a co-occurrence matrix (GLCM) include entropy (randomness of the matrix), energy/angular second moment (pixel repetition/orderliness and measures the homogeneity of an image), homogeneity (uniformity of co-occurrence matrix), dissimilarity (measurement of how different each element in the matrix is), and correlation (measurement of gray-tone linear dependencies). Another method to derive second-order statistics is the run-length matrix (RLM), which analyzes texture in a specific direction. A run is a length of consecutive pixels with the same gray-level intensity in a preset direction. The relationships between the run lengths give rise to texture. Fine texture has more short runs with similar gray-level intensities, whereas coarse texture has more long runs with different gray-level intensities. Some of the RLM parameters include short-run emphasis (SRE; measures distribution of short runs in an image), long-run emphasis (LRE), gray-level non-uniformity (GLNU; measures similarity of gray-level values; GLNU is small if variation is less), and run length non-uniformity (RLNU; measures the similarity of run lengths; RLN is small if run lengths are similar). Higher-order statistics are calculated using neighborhood gray-tone-difference matrices, which examine the spatial relationship among three or more pixels and are thought to closely resemble the human experience of the image [14, 15]. This is calculated using the neighborhood gray-tone-difference matrix (NGTDM). Examples of higher-order statistics include contrast (number of local variations within the image), coarseness (measurement of edge density), and busyness (measurement of spatial rate of gray-level change). The application of filters such as Laplacian of Gaussian bandpass filters in statistical-based texture analysis of an image allows the extraction of specific structures corresponding to the width of the filters. Lower filter values (filter 0.5-1.0) will highlight structures with fine textures, and higher filter values highlight structures with medium (filter 1.5-2.0) and coarse (filter 2.5) textures in the filtered image.

Model-based approaches represent texture using sophisticated mathematical models such as fractal analysis. Fractal analysis is a form of pattern or geometric recognition. The fractal dimension is a measurement of the irregularity or roughness of a surface [13, 15]. Hence, the greater the fractal dimension is, the rougher the texture.

Transform-based methods, such as Fourier, Gabor, and wavelet transforms, analyze texture in a frequency or the scale space. Fourier transform analyzes the frequency content without spatial localization and hence is not often used. Gabor transform is essentially a windowed-Fourier transform derived by the introduction of Gaussian function, which then allows for frequency and spatial localization but is limited by its single filter resolution. This problem is overcome by wavelet transform, which uses multiple channels tuned to different frequencies [15] (Appendix).

**Fig. 2:**
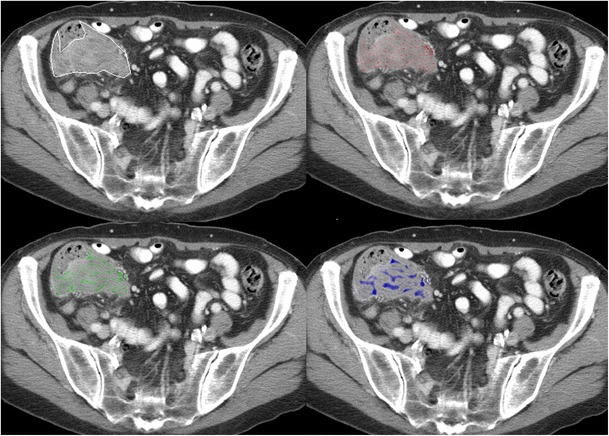
Texture analysis of contrast-enhanced CT images of a colon cancer with the application of different filters highlighting fine, medium, and coarse textures

**Fig. 3:**
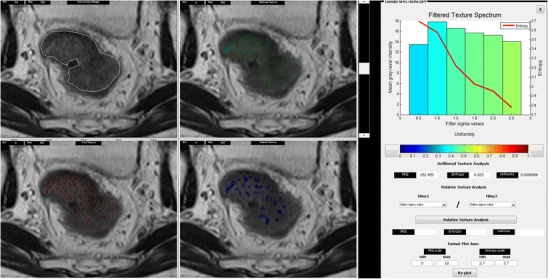
Texture analysis of a T2-weighted MRI image of rectal cancer

Texture analysis is not a new technique and has been studied for medical imaging since 1973, when applied to radiographs, and subsequently to ultrasound [16–18]. More recently, texture analysis has been applied to CT (Figs. 2, 3) and MRI, with an increasing number of PET studies [10, 19–31]. In oncological imaging, texture analysis is re-emerging as a potential tool with an increasing number of published studies. A major advantage of texture analysis is that information is maximized from clinical images without the need for additional acquisitions. Studies have focused in several areas: feasibility, technical optimization, validation, and potential clinical applications. This article reviews the the current evidence for texture analysis of CT, MRI, and PET/CT images and the clinical potential in the field of oncology.

## Texture analysis of computed tomography images

Although much of the heterogeneity visible on CT may represent photon noise, which can mask any underlying biological heterogeneity, texture analysis of CT images has been shown to be feasible by reducing the effect of photon noise while enhancing biological heterogeneity [6, 32]. A few studies have compared texture features with other imaging and biological parameters (Table 1) [33–35], providing early evidence of potential correlates of CT texture. For example, coarse texture features may reflect the underlying vasculature as defined by CD34 [35]. Nevertheless, further research in this area is still needed.

**Table 1:** Studies correlating texture features to other imaging and biological parameters

Cancer type	Features investigated	Correlate	Author, year
Esophagus	Non-contrast CT. Coarse texture uniformity (*r* = −0.754, p < 0.0001)	SUV_mean_	Ganeshan et al., 2012 [33]
	Entropy (*r* = 0.748, *p* = 0.0001)		
NSCLC	Contrast-enhanced CT	SUV_max_	Al-Kadi et al., 2008 [12]
	Fractal dimension		
NSCLC	Non-contrast CT	SUV_max_	Ganeshan et al., 2008 [44]
	Coarse texture		
	Uniformity (*r* = −0.52, *p* = 0.03)		
	Entropy (*r* = 0.51, *p* = 0.03)		
NSCLC	Contrast-enhanced CT	Histological: CD34 and pimonidazole	Ganeshan et al., 2012 [35]
	Medium and coarse texture; SD^a^ (*r* = −0.579, *p* < 0.001) (*r* = 0.591, *p* < 0.001)		

^a^*SD* standard deviation of the histogram

To date, studies that have been performed have focused in several areas, where the addition of texture to current methods may improve the detection, diagnosis, characterization, and response assessment (Table 2) [5, 32–34; 36–43]. By highlighting certain features within a lesion of interest, texture analysis has the ability to improve assessment beyond direct visual analysis by a radiologist.

**Table 2:** Studies investigating the use of CT texture analysis in diagnosis, treatment response assessment, and as a prognostic tool

Diagnosis and characterisation	Method	Study findings	Author, year
Diagnosis			
Lung			
Pulmonary nodules	Fractal analysis	3D fractal dimension was higher in organizing pneumonias/tuberculomas than carcinomas/hamartomas (p < 0.001) and higher in adenocarcinomas than squamous cell (p < 0.05)	Kido et al., 2002 [37]
Bronchoalveolar carcinoma vs. non-bronchoalveolar carcinoma	Fractal analysis	Fractal dimension higher for bronchoalveolar carcinomas (2.38 ± 0.05/2.16 ± 0.01) than non- bronchoalveolar carcinomas (2.19 ± 0.05/2.06 ± 0.01 internal/peripheral; p < 0.0001)	Kido et al., 2003 [36]
Lung cancer	Fractal analysis	Fractal dimension was higher for stage III and IV cancers than stage I (2.046 vs. 1.534). 83.8 % of stage IV tumors were classified as aggressive with a threshold of 1.913	Al-Kadi et al., 2008 [5]
Liver			
Hepatic tumors	Texture analysis	Autocovariance function differed between malignant (HCC and colorectal metastases) and benign lesions. Sensitivity of 75.0 % and specificity of 88.1 % were achieved with the proposed diagnostic system	Huang et al., 2006 [38]
GI tract			
Colorectal cancer	Fractal analysis	Fractal dimension and abundance were higher in colon cancer than normal bowel: mean (SD) 1.71(0.07) vs. 1.61(0.07) for dimension and 7.82(0.62) vs. 6.89 (0.47) for abundance (*P* ≤ 0.001)	Goh et al., 2007 [8]
Colorectal cancer	Texture analysis	Fractal dimension is higher for metastatic nodes	Cui et al., 2011 [39]
Brain			
Glioma	Texture analysis	Coarse texture entropy >5.2 had a sensitivity and specificity of 76 % and 82 %, respectively; uniformity <0.025 had a sensitivity and specificity of 64 % and 95 %, respectively, for high-grade tumors	Skogen et al., 2011 [40]
Response assessment			
Metastatic renal cell carcinoma	Texture analysis	Percentage change in coarse texture uniformity of ≤ −2 % after 2 cycles of TKI correlated with shorter time to progression	Goh et al., 2011 [41]
Prognosis assessment			
Liver texture in patients with colorectal cancer but no known metastases	Texture analysis	Coarse texture entropy correlated with hepatic perfusion index (*r* = −0.503978, *p* = 0.007355) and survival (*r* = 0.489642, *p* = 0.009533). Hypothesized texture features may reflect vascular changes associated with micrometastases. Entropy <2.0 identified patients who died with 100 % sensitivity, 65 % specificity	Ganeshan et al., 2007 [42]
Colorectal cancer metastases	Texture analysis	Uniformity at texture ratios of 1.5/2.5 and 2.0/2.5 were significant OS prognostic factors (*p* < 0.005)	Miles et al., 2009 [43]
Liver texture in patients with colorectal cancer	Texture analysis	Fine texture entropy of ≤0.0807 between 26–30 s after contrast injection highlighted node-positive patients with 100 % sensitivity, 71 % specificity. HPI did not vary significantly between node-negative and -positive patients	Ganeshan et al., 2011 [32]
Esophageal cancer	Texture analysis	Unenhanced CT component of PET-CT	Ganeshan et al., 2012 [33]
		Greater heterogeneity in higher stage tumors. Coarse uniformity was a significant OS prognostic factor (*OR* = 4.56, 95 % CI 1.08–18.37, *p* = 0.039)	
NSCLC	Texture analysis	Coarse texture uniformity <0.624 was a poor prognostic factor	Ganeshan et al., 2011 [34]

### Diagnosis and characterization

**Fig. 4:**
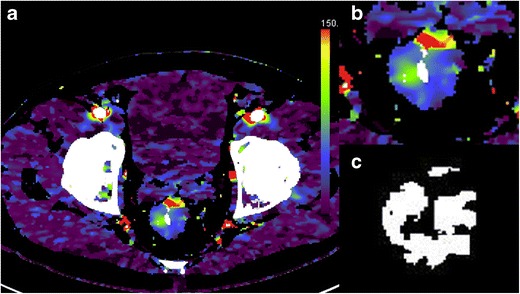
Dynamic contrast-enhanced CT (perfusion CT) blood flow parametric map (**a**); 2D image (**b**); segmented and thresholded image (**c**) for fractal analysis

Several studies have applied various texture analysis methods to improve lesion characterization based on the hypothesis that there are texture differences between benign and malignant lesions. In common, these studies have found that there is greater heterogeneity and higher fractal dimension in tumors than benign lesions, which has the potential to contribute to the computer-aided diagnosis (CAD) of lung or liver lesions. For example, Huang et al. investigated the role of autocovariance function of unenhanced CT images for classifying liver lesions as malignant (80 lesions) or benign (84 lesions). The 2D normalized autocovariance coefficient is a statistical-based texture feature that measures the interpixel correlation within an image. The authors found that its accuracy was 81.7 % for differentiating malignant from benign lesions, but overlap precluded differentiation of primary and secondary tumors [38]. Kido et al. attempted to classify small lung lesions in high-resolution CT images in two separate studies using fractal analysis [36, 37]. In the first study, they evaluated the use of fractal analysis in differentiating benign from malignant lung tumors by comparing either 2D binary or 3D gray-level intensity mapping of the fractal dimension in biopsy-proven lesions [37]. Benign hamartomas (*n* = 23) had smaller 2D fractal dimensions (1.17 ± 0.05) compared to bronchogenic carcinomas (*n* = 70) (1.23 ± 0.07), organizing pneumonias (*n* = 13) (1.22 ± 0.07), and tuberculomas (*n* = 11) (1.25 ± 0.07) (*p* < 0.05). However, carcinomas (2.10 ± 0.11) and hamartomas (2.12 ± 0.06) had smaller 3D fractal dimensions compared to organizing pneumonias (2.29 ± 0.17) and tuberculomas (2.25 ± 0.08) (*p* < 0.0001). The 3D fractal dimension also differentiated adenocarcinomas (*n* = 61) from squamous cell carcinomas (*n* = 9) [37]. In the second study, the authors found that bronchoalveolar carcinomas (*n* = 30) had greater fractal dimensions compared to non-bronchoalveolar carcinomas (*n* = 40) [36]. Al-Kadi et al. compared fractal analysis of dynamic contrast-enhanced CT (wash-in/wash-out) images. The mean fractal dimension was higher in aggressive/advanced stage tumors compared to non-aggressive/early stage tumors with 83.3 % accuracy for distinguishing between these two groups [5]. Goh et al. found that the fractal dimension and fractal abundance were higher for colorectal tumors compared to normal colon, mean fractal dimension, and abundance (standard deviation): 1.71 (0.07) and 7.82 (0.62), respectively, for tumor and 1.61 (0.07) and 6.89 (0.47), respectively, for normal colon (*p* ≤ 0.001) (Fig. 4) [8]. Cui et al. studied 220 nodes in colorectal cancer and suggested that CT texture features of malignant and benign nodes differed, with greater heterogeneity noted in malignant nodes with a predictive accuracy of 88 % [39]. In a study that included 44 patients with gliomas, Skogen et al. found that coarse texture entropy and uniformity could distinguish between low- and high-grade tumors. Entropy >5.2 had a sensitivity and specificity of 76 % and 82 %, respectively; uniformity ≤0.025 had a sensitivity and specificity of 64 % and 95 %, respectively, for high-grade tumors [40].

### Therapy response assessment

**Fig. 5:**
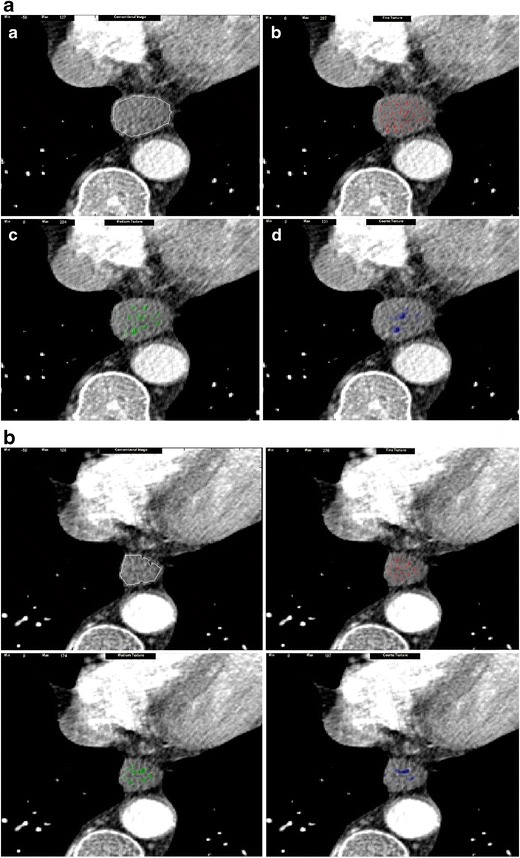
Changes in texture features of esophageal cancer following neoadjuvant chemotherapy: baseline (**a**) and following chemotherapy (**b**). An increase in homogeneity is noted with treatment

**Fig. 6:**
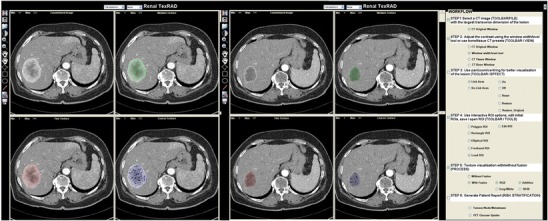
Changes in texture features of metastatic renal cancer following two cycles of a tyrosine kinase inhibitor: baseline (*left*) and following therapy (*right*). An increase in homogeneity is noted with treatment

**Fig. 7:**
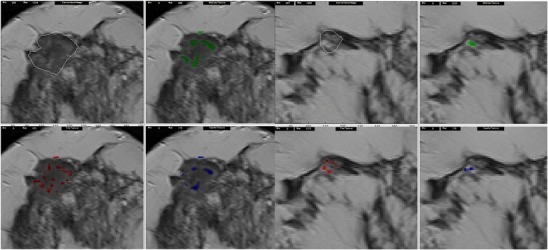
Changes in breast tumor texture following neoadjuvant chemotherapy on T2W-weighted MRI. Tumor shrinkage and an increase in homogeneity are noted following completion of chemotherapy (*right*)

To date, few studies have assessed the potential of texture analysis for response assessment (Figs. 5, 6, 7). Goh et al. investigated the potential of texture analysis to improve response assessment in renal cell cancer metastases treated with tyrosine kinase inhibitors. This study found that texture analysis was a better predictor of response than current response assessment methods based on size and/or enhancement change (RECIST and modified Choi). The percentage changes from baseline values of texture features after two cycles of TKI therapy for metastatic renal cancer were correlated with measured time to progression. Using a threshold identified by ROC analysis, the percentage change of −2 % or less from baseline for uniformity at a filter value of 2.5, disease-free survival was significantly better in the group with greater than −2 % change in uniformity (*p =* 0.008) and performed better than standard response assessment after two cycles of TKI therapy [41].

### Relationship with clinical outcome: A potential prognostic biomarker?

Several studies have assessed the potential of texture analysis to improve the prognostic information of current imaging and confirm the hypothesis that greater tumor heterogeneity is an indicator of poor clinical prognosis [33, 42–44]. Texture analysis as a post-processing tool may complement the prognostic information obtained from standard imaging.

Ganeshan et al. found that coarse texture entropy on CT in seemingly normal liver of patients with colorectal cancer correlated with patient survival and postulated that this may be related to micrometastasis formation [42]. The authors also investigated patients with colorectal metastases. They normalized finer texture values (filter 1.0-2.0) to the corresponding texture values obtained from the coarsest filter (filter 2.5) to account for the contribution to the overall texture by the different texture features obtained from different levels of image filtration. It was found that the uniformity of surrounding ‘normal’ liver at texture ratios of 1.5/2.5 and 2.0/2.5 predicted for survival, potentially related to differences in vascularization and shunting related to the presence of metastases [43]. The same group also assessed the texture of dynamic contrast-enhanced CT of the liver in node-negative and -positive non-metastatic colorectal cancer. Uniformity and entropy were significantly different between the node-positive and -negative patients and greatest for fine texture entropy between 26 and 30 s following injection of intravenous contrast in comparison with the hepatic perfusion index, which was not significantly different between the two groups [32]. In a study of 54 patients with non-small-cell lung cancer (NSCLC) undergoing PET-CT staging, a heterogeneous texture on the non-contrast-enhanced CT component of the PET-CT was a predictor of poorer survival; in particular, patients with coarse texture uniformity <0.624 did not survive more than 2.5 years [44]. Similarly, in a study of 21 patients with primary esophageal cancer undergoing PET-CT staging, advanced stage tumors demonstrated greater heterogeneity at filter values 1.5–2.0. Survival was also poorer for more heterogeneous tumors, particularly for coarse texture uniformity <0.8477 (odds ratio = 4.45, 95 % CI 1.08–18.37, *p* = 0.039) [33].

## Texture analysis of magnetic resonance imaging

Texture analysis has also shown promise in MRI. The majority of literature over the past 10 years has been directed toward lesion detection and lesion classification, for example, breast, brain, liver, and prostate (Table 3) [19–23, 26, 45–48].

**Table 3:** Studies investigating the use of MRI texture analysis in diagnosis, treatment response assessment, and as a prognostic tool

Diagnosis and characterization	Method	Study findings	Author, year
Diagnosis			
Breast			
Simulated microcalcification	Texture analysis	Successful automatic detection of localized blurring was achieved (*sensitivity* = 89 %–94 %; *specificity* = 99.7 %―100 %; *PPV* = 74 %–100 %; *NPV* = 99.9 %–99.9 %)	James et al., 2001 [45]
Breast cancer	Texture analysis	A combination of textural analysis (second-order statistics, e.g., contrast, sum entropy, entropy), lesion size, time to maximum enhancement, and patient age allowed for a diagnostic accuracy of 0.92 ± 0.05	Gibbs et al., 2003 [46]
Breast lesion	Texture analysis	The classification performance of volumetric texture features (second-order statistics) is significantly better than 2D analysis	Chen et al., 2007 [47]
Breast cancer	Texture analysis	The 4D texture analysis (using second-order statistics) achieved a performance comparable to human observers	Woods et al.,2007 [48]
Invasive lobular and ductal breast cancer	Texture analysis	Investigated the use of first-order statistics, second-order stastistics obtained from GLCM, RLM, autoregressive model, and wavelet transform. All parameters distinguished healthy from cancerous tissue although GLCM performed better. 80 %–100 % of accuracy in differentiating ductal from lobular cancers, particularly complexity and entropy	Holli et al., 2010 [22]
Brain			
Glioneuronal tumor	Texture analysis	The combination of DCE-MRI and MRI textural analysis (second-order statistics—GLCM and RLM) provide optimal differentiation between glioneuronal tumors and gliomas in vivo	Eliat et al., 2012 [19]
Brain tumors—metastases, meningiomas, gliomas (grade II and III), glioblastomas	Texture analysis	Metastases were successfully distinguished from gliomas (*accuracy* = 85 %; *sensitivity* = 87 %; *specificity* = 79 %) as well as high-grade from low-grade neoplasms (*accuracy* = 88 %; *sensitivity* = 85 %; *specificity* = 96 %) using Gabor transform texture analysis	Zacharaki et al., 2009 [23]
Prostate			
Prostate cancer	Fractal analysis	The combination of fractal and multifractal features was more accurate than classical texture features in detecting cancer and was more robust against signal intensity variations	Lopes et al., 2011 [21]
Prostate cancer	Fractal analysis	Both fractal analyses offered promising quantitative indices for prostate cancer identification, with histogram fractal dimension offering a more robust diagnosis than texture fractal analysis (correlation coefficient of *c* = 0.9905 vs. *c* = 0.9458, respectively)	Lv et al., 2009 [49]
Liver			
Liver cysts and hemangiomas	Texture analysis	Texture analysis (first-order, second-order statistics and wavelet transform) was successfully used to classify focal liver lesions on zero-fill interpolated 3.0-T MR images	Mayerhoefer et al., 2010 [20]
Response assessment			
Breast	Texture analysis	Second-order statistics extracted from parametric maps that reflect lesion washout properties discriminate malignant from benign tumors better than textural features extracted from either first post-contrast frame lesion area or from parametric map reflecting lesion initial uptake. Angular second moment and entropy were most discriminative	Karahaliou et al., 2010 [26]
Lymphoma	Texture analysis	Texture analysis [first-order, second-order statistics (GLCM and RLM), autoregressive model and wavelet transform] was able to classify NHL lesions undergoing chemotherapy based on changes following treatment	Harrison et al.,2009 [27]
Liver metastases	Fractal analysis	Tumor heterogeneity as assessed by fractal dimension predicted tumor shrinkage in response to bevacizumab and cytotoxic chemotherapy in colorectal liver metastases	O’Connor et al., 2011 [28]

### Diagnosis and characterization

As with CT, MRI studies have found that texture features may differ between benign and malignant lesions and may have potential in CAD. In the breast 2D co-occurrence matrix features of dynamic contrast-enhanced MRI images and signal enhancement ratio maps, 3D and 4D features may distinguish between benign and malignant breast lesions: 4D techniques may achieve a performance similar to human observers (AUC 0.99) [26, 46–48]. Holli et al. have investigated differences in texture between invasive lobular carcinoma (ILC) and invasive ductal carcinoma (IDC), two common but distinct types of breast cancer, using different texture methods. In this study, co-occurrence matrix features were significantly different between ILC and IDC, allowing differentiation between these two histological subtypes, and were superior to the other texture methods applied including histogram analysis, run-length matrix, autoregressive model, and wavelet transform [22].

In the brain, studies have found that texture features of MRI including dynamic contrast-enhanced (DCE) sequences may distinguish between types of tumors [19, 23]. In the first study by Eliat et al., the addition of MRI texture analysis to dynamic contrast-enhanced MRI (DCE-MRI) was able to discriminate glioblastoma multiforme (GBM) from malignant glioneuronal tumors (MGNT) [19]. This study analyzed the use of both first-order and second-order statistics, which included GLCM and RLM methods. This study found that the addition of second-order statistics such as run-length non-uniformity, gray-level non-uniformity, angular second moment, and entropy to the findings from DCE-MRI had 100 % negative predictive value, 79 % positive predictive value, 100 % sensitivity, and 62 % specificity in differentiating MGNT from GBM. Another group developed a computer-assisted classification method combining conventional MRI and perfusion MRI texture analysis using Gabor transform and its implementation as a diagnostic tool [23]. When the method was applied to 102 different brain tumors, including metastasis (*n* = 24), meningiomas (*n* = 4), grade II gliomas (*n* = 22), grade III gliomas (*n* = 18), and glioblastomas (*n* = 34), the accuracy, sensitivity, and specificity achieved by this method were 85 %, 87 %, and 79 %, respectively, for discrimination of metastases from gliomas and 88 %, 85 %, and 96 % for discrimination of high-grade (grades III and IV) from low-grade (grade II) neoplasms.

In the liver, an exploratory study on unenhanced T1- and T2-weighted MRI showed that it was feasible to use texture analysis to classify benign cysts and hemangiomas, though with up to 25 % misclassified [20]. This study used first- and second-order statistics (GLCM and RLM) and also the wavelet transform method to derive texture parameters that were then selected based on their discriminative value in differentiating cysts from haemangiomas and subsequently used in the computer-assisted classification algorithm. Two prostate studies have shown the potential of fractal features in distinguishing between benign and malignant disease with histological confirmation [21, 49]. For example, Lv et al. investigated the use of the texture fractal dimension (TFD) and histogram fractal dimension (HFD) based on the box-counting method and histogram fractal analysis of the intensity distribution, respectively. The mean and standard deviations of TFD and HFD for cancerous and non-cancerous lesions were significantly different (TFD: 2.76 ± 0.11 vs. 2.81 ± 0.15, *p* = 0.035; HFD: 1.23 ± 0.05 vs. 1.42 ± 0.09, *p <* 0.001). The authors also showed that an area under the ROC curve of 0.96 could be achieved for the histogram fractal dimension in a cohort of 55 patients who had diagnoses confirmed by ultrasound-guided biopsy [49].

### Therapy response assessment

Studies of response assessment have shown that assessment of heterogeneity is feasible (Fig. 5), may augment response assessment, and is a predictor of response [24, 27, 28]. Harrison et al. demonstrated that MRI texture appearances change during treatment in 19 patients with non-Hodgkin lymphoma who were imaged with T1 and T2W MRI at three time points: at staging, and after the first and fourth cycle of chemotherapy [27]. Alic et al. examined the role of MRI texture in response prediction following isolated limb perfusion in unresectable soft tissue sarcoma of the extremities. They showed that responding tumors demonstrated high coherence in the pre-treatment MRI, a texture parameter that measures how spatially close the high intensity voxels are to each other [24]. Similarly, O’Connor et al. demonstrated in 10 patients with 26 liver metastases from colorectal cancer treated with bevacizumab and FOLFOX-6 chemotherapy that fractal measures derived from pre-treatment DCE-MRI were associated with tumor response [28].

## Texture analysis of positron emission tomography (PET)

At present, only a few studies have investigated the potential of PET texture analysis. These studies have focused on its prediction of outcome and potential for radiotherapy planning.

### Prediction of outcome

To date, three studies have been published. Eary et al. showed that image heterogeneity as assessed by a new method using a heterogeneity variable (HET), which was defined as the percentage of variability in the voxel-level FDG uptake compared to an ‘ideal’ ellipsoidal upake pattern, was a valid prediction method in patients with sarcoma. An increase of 6.5 % in heterogeneity was associated with a 65 % increase risk of death, the risk being higher in patients with high-grade disease [50]. El Naqa et al. demonstrated that various first- and second-order statistical textural features are useful in predicting outcome in head and neck (*n* = 9) and cervical cancer (*n* = 14). The authors combined the various first- and second-order statistics (energy, contrast, local homogeneity, and entropy) with features indicating the shape of the tumors in a linear regression model to predict treatment response to chemoradiation. These methods achieved an AUC of 0.76 and 1.0 for the cervix and head and neck cohorts, respectively [30]. Tixier et al. have investigated its clinical application in 41 patients with esophageal cancer treated with chemoradiation and shown that baseline FDG PET texture is a sensitive predictive marker. They found that local (i.e., entropy and homogeneity) and regional (i.e., size and intensity variabilities) texture parameters performed better than standard SUV measurements in identification of responders from non-responders following chemoradiation. The sensitivity, specificity, and AUC for SUVmax were 53 %, 73 %, and 0.59 compared to 73 %, 88 %, and 0.89 for local homogeneity in identifying responders [31].

### Potential for radiotherapy planning

Yu et al. have assessed whether first-order, second-order, and higher-order statistics in FDG PET-CT co-registered images can differentiate between normal and abnormal nodes to assist radiotherapy target planning. Abnormal nodes were found to be more heterogeneous than normal tissues in PET images and, of interest, have higher uniformity in CT images [51]. It could be that the accuracy of texture analysis based on non-contrast enhanced CT scan is lower than PET texture or that there was indeed a true difference between CT and PET textures when abnormal tissues are compared to the normal tissues. However, the authors found that a combination of PET and CT textures, particularly second-order and higher-order statistics, had higher discriminative power. This group subsequently developed an automated radiotherapy volume delineation software (“COMPASS”) based on their findings from the initial study. They studied its use in ten patients with head and neck cancer by comparing this to three PET segmentation methods: threshold SUV value of 2.5, threshold of 50 % maximal intensity and signal/background ratio, as well as tumor volume delineation by three independent radiation oncologists. This study found that automated texture-based segmentation correlated better with tumor delineation by oncologists compared to PET segmentation [52].

## Technical challenges for clinical implementation of texture analysis

Although studies so far have shown clinical promise, there are still technical considerations to contemplate, particularly the effect of image acquisition, image quality, texture methods and software platforms on parameter values, the need for harmonization of acquisitions, and a standardized analysis approach for clinical application [5, 12, 32, 53–56]. Each of the modalities brings different challenges to texture analysis. With PET, images are of a lower spatial resolution than CT or MRI. Although there is no specific limit to the size of the lesion that is amenable to this analysis, small tumors such as nodes, which are below the spatial resolution of PET scans, may not be suitable for this technique. With MRI, scanner and sequence acquisition parameters have a greater non-linear influence on signal intensity and quantification of heterogeneity in comparison with CT or PET, thus requiring stringent quality control and physics input.

To date, many studies have focused on a limited tumor area, such as the largest cross-sectional area, rather than the whole tumor volume. Intratumoral heterogeneity is likely to be greater in the whole tumor as compared to a limited region; hence, this could dilute the diagnostic and prognostic value of texture analysis. With region-of-interest delineation around a tumor, this has the potential to introduce inter- and intraobserver variability. If a standardized automated ROI propagation is used, non-tumor areas may be included in the analysis of the pixel values, which may confound the results obtained.

However, the potential impact of this methodical difference on clinical findings is largely unexplored. At present, the reproducibility of texture analysis has yet to be established widely, although a few studies have begun to address issues related to image acquisition and image quality, and their effect on texture analysis [32, 53–56]. For example, Sanghera et al. have demonstrated that the reproducibility of fractal analysis in two scans performed 24 h apart by a single reader is good, with a mean difference (95 % limits of agreement) of −0.024 (−0.212 to 0.372) and −0.355 (−0.869 to 1.579) for 2D fractal dimension and fractal abundance, and −0.024 (−0.307 to 0.355) and −0.043 (−1.154 to 1.239) for 3D fractal dimension and fractal abundance, respectively [9]. Good interobserver variability is also observed with a mean difference of 0.030 (95 % limits of agreement −0.143 to 0.204) and −0.073 (−0.823 to 0.676) for 2D and 3D fractal dimensions, and −0.073 (−0.823 to 0.676) and −0.044 (−0.139 to 0.052) for 2D and 3D fractal abundances, respectively. For therapeutic assessment where repeated imaging is performed, it may be appropriate to use the same scanner and acquisition parameters to ensure consistency. Similarly, if contrast-enhanced images are used, contrast agent administration should be consistent to minimize variability in gray level intensity related to differences in contrast agent administration or dose. Use of a ‘texture phantom’ would also allow calibration of imaging systems within a multicenter setting. Further work in this area is still needed.

The implementation of texture analysis into the routine clinical workflow will remain a challenge. Although patients are not required to undergo any additional imaging, as texture analysis is a post-processing step that can be performed on existing DICOM format images, at present, such analysis and software remain as research tools, with few commercially available options.

## Conclusions

Although texture analysis is not a new technique, there has been resurgent interest in the assessment of tumor heterogeneity, particularly for CT, MRI, and PET, in the last 10 years, albeit in relatively small studies. Nonetheless, it is showing promise in the diagnosis and characterization of tumors, response assessment, and as a predictive biomarker, which should be explored further in larger prospective studies. Texture analysis maximizes the information obtained from current standard images and can be implemented into the clinical workflow. With further efforts to refine its applications and direct standardization, this technique has the potential to develop into a valuable clinical tool in oncologic imaging in the future.

## Appendix

### Statistical-based approaches applied to CT images

An approach that has been used in recent publications is the application of a Laplacian of Gaussian (LoG) bandpass filter to highlight and enhance different spatial scales between fine and coarse texture (filter value = 1.0 to 2.5) [1].

The two-dimensional (2D) Gaussian distribution *G* is given by:

E1

where *x*, *y* are the spatial coordinates of the image matrix and σ is the standard deviation of the Gaussian distribution.

The 2D Gaussian distribution effectively blurs the image, wiping out all structures at scales much smaller than the sigma of the Gaussian. This distribution has the desirable characteristics of being smooth and localized in both the spatial and frequency domains, and is therefore less likely to introduce any changes to the original image. The Gaussian distribution highlights only texture features of a particular scale. A fine scale (< 4 pixels) enhances parenchyma, while a medium-to-coarse scale (6–12 pixels) enhances the underlying vasculature.

The reason for using the Laplacian (∇^2^) is that it is the lowest-order orientation-independent (isotropic) differential operator and inherently has less computational burden and can be used to detect intensity changes in an image that correspond to the zero crossings of the filter. ∇^2^  *G* is the Laplacian of Gaussian filter, a circularly symmetric, Mexican-hat-shaped filter whose distribution in the 2D spatial domain is given by

E2

From the mathematical expression of this circularly symmetric filter at different filter values, the number of pixels representing the width between the diametrically opposite zero-crossing points in this filter can be calculated. The width of the filter at different filter values is obtained by evaluating the Laplacian of the Gaussian spatial distribution along the *x* and *y* directions. The lower the filter value, the smaller is the filter width in the spatial domain and the larger is the pass-band region of the filter in the frequency domain, highlighting fine details or features in the filtered image in the spatial domain. Similarly in the spatial domain, a higher filter value allows coarse features to be highlighted in the filtered image.

Filtration can be done in the spatial or frequency domain. In the spatial domain, the filter mask is convolved with the image, which involves intensive computation. It is more efficient to use the filter in the frequency domain, as convolution of the filter mask and the image in the spatial domain is equivalent to multiplication of the Fourier transforms of the filter mask and image in the frequency domain. The inverse Fourier transform of the filtered spectrum gives the resultant filtered image in the spatial domain. Also, the accuracy of this filtering operation is improved when used in the frequency domain, as the quantization errors arising from the convolution of the filter, especially for small σ values in the spatial domain, would distort the image.

Quantification of CT texture following filtration is typically performed for a specified region of interest (e.g., the largest tumor cross-sectional area) or for the whole tumor. Thresholds can be applied to the original CT image. In the case of rectal or lung tumors, this may be to exclude surrounding air by removing any pixels with attenuation values below −50 HU from the analysis. The same ROI or VOI is applied at all filter scales.

Typical parameters derived from the histogram analysis include the kurtosis, skewness, and standard deviation of the pixel distribution histogram, mean gray level intensity, entropy, and uniformity. Kurtosis (or the magnitude of pixel distribution), skewness (or the skewness of the pixel distribution), and the standard deviation of the pixel distribution describe the shape of the histogram representing the peak, asymmetry, and gray-level variation within the lesion. Entropy is a measure of texture irregularity, while uniformity reflects the distribution of gray levels within the tumor.

Higher entropy, lower uniformity, higher standard deviation, higher kurtosis, and positive skewness are thought to represent increased heterogeneity and portend poorer prognosis [2–4].

E3

E4

E5

E6

E7

Where:

is the mean value within *R*, *R* is the ROI within the image *a(x,y)*, *n* is the total number of pixels in *R*, *I* is the pixel value (between *I* =1 to *k*) in *R*, and *p(I)* is the probability of the occurrence of that pixel value.

### Autocorrelation model

The autocorrelation coefficient denotes the interpixel correlation within an image. This coefficient is modified to a mean-removed version to generate similar autocorrelation features with different brightness but similar texture called the autocovariance coefficient [5]. The autocovariance coefficient between pixel (*i,j*) and pixel (*i +* ∆*m, j* + ∆*n*) in an image of size *M* x *N* is given by:

E8

E9

where ˉ*f* is the average value of *f*(*x, y*).

### Model-based approaches applied to CT images

#### Fractal analysis

Fractal analysis provides a means of assessing structural geometry. Fractal measures such as the fractal dimension (a measure of how an object fills space), fractal abundance (a measure of the volume of space filled), and lacunarity (a measure of the structural heterogeneity within an object) inform about different aspects of the spatial pattern of the tumor vasculature, providing a measure of its spatial heterogeneity. Fractal dimension and fractal abundance may be calculated using a 2D square and 3D cube, the box counting method, with multiple grid offsets for all possible box start locations, based on the following equation:

E10

where L is the box size, N_L_ is the number of boxes of size L needed to cover the object being studied, and D is the fractal dimension. By plotting a log-log plot of N_L_ versus L, fractal dimension (FD) can be obtained from the slope, and fractal abundance (FA) or log K can be obtained from the y-intercept of the straight portion of the curve [6]. The 2D and 3D techniques have been validated using simple and complex structures of known fractal dimension, e.g., Sierpinski gasket and carpet with multiple iterations, and shown to be reproducible [7].

Lacunarity can be derived using a 2D and modified 3D gliding box method defined by the equation:

E11

where Λ represents lacunarity, r represent box size, s represents the number of occupied sites within a box size, and Q is the probability distribution (representing the frequency distribution of the total number of occupied sites for a box size r over the total number of boxes of size r) [8].

#### Transform-based approaches applied to CT images

The pixel pattern in a 2D image has a unique frequency pattern in a specific spatial scale of the image [9, 10]. If the gray-level values change quickly, it is deemed to have a high spatial frequency. If the gray level values vary slowly such that there is very little variation within an image, it is considered to have a low spatial frequency. This is dependent on the scale used to analyze the image. For example, if a large scale is used, then there is less variation observed compared to the greater variation and details seen with a smaller scale.

#### Fourier transform

Fourier transform is useful in the analysis of global frequency content but is without time/space localization [11]. The window Fourier transform of a 1D signal *f (x)* is given by:

E12

#### Gabor transform

Gabor transform introduces the time dependency element into Fourier analysis by multiplying it with a Gaussian function [i.e., window function *w(x)* becomes Gaussian] [12]. The time-frequency resolution of the Gabor transform is fixed throughout the time-frequency plane [12]. Gabor output is also non-orthogonal; thus, the resultant texture features may have significant correlation [13].

#### Wavelet transform

Multiresolution analysis such as wavelet transform uses multiple channels tuned to different frequencies (i.e., the window function varies). Wavelet transform was first introduced by Mallat [14]. It decomposes an image by using spatially oriented frequency filters but requires intensive computation. Wavelets are considered as a family of functions derived from translations and dilations of a single function known as the “mother wavelet” Ψ(t). Wavelets are defined by:

E13

where *a* is the scaling parameter and measures degree of compression, and *b* is the translation parameter that indicates the time location of the wavelet. If │*a*│ < 1 indicates the compressed version of the mother wavelet and is of higher frequency, then │*a*│ > 1 corresponds to lower frequencies [15].

Table 4. Definitions of statistical texture features

**Table:**

Order of statistics	Texture features	Definitions
First-order statistics	Mean gray-level intensity	Average pixel value, i.e., intensity/brightness of a region
	Standard deviation	Variation from mean gray-level value
		SD is small if image is homogenous
	Uniformity	Uniformity of gray-level distribution
	Entropy	Irregularity of gray-level distribution
	Kurtosis	Flatness of histogram
	Skewness	Asymmetry of histogram
Second-order statistics	Local entropy	Measures randomness in image
		Higher entropy indicates greater randomness
	Local homogeneity	Measures closeness of distribution of gray-level values in the matrix (GLCM) to the GLCM diagonal
	Angular second moment (ASM)/energy	Measures homogeneity in an image. Higher value indicates greater uniformity of gray-level values in a matrix
	Dissimilarity	Measurement of how different each element in the matrix is
	Correlation	Measures gray-tone linear dependencies
Higher-order statistics	Coarseness	Measures the edge density
		Finer texture has higher edge density
	Busyness	Measures spatial rate of gray-level change
	Contrast	Difference moment of the matrix, measures local variations and spread of matrix values
		High contrast indicates greater local variation, i.e., more heterogeneous
	Complexity	Measures the amount of information in an image (gray-level intensities, number of sharp edges)

**References**

1. Ganeshan B, Miles KA, Young RC, Chatwin CR (2007) Hepatic entropy and uniformity: additional parameters that can potentially increase the effectiveness of contrast enhancement during abdominal CT. Clin Radiol, 62(8):761–768.
2. Ganeshan B, Abaleke S, Young RC, Chatwin CR, Miles KA (2010) Texture analysis of non-small cell lung cancer on unenhanced computed tomography: initial evidence for a relationship with tumour glucose metabolism and stage. Cancer Imaging, 10:137–143.
3. Miles KA, Ganeshan B, Griffiths MR, Young RC, Chatwin CR (2009) Colorectal cancer: texture analysis of portal phase hepatic CT images as a potential marker of survival. Radiology, 250(2):444–452.
4. Kato H, Kanematsu M, Zhang X, et al. (2007) Computer-aided diagnosis of hepatic fibrosis: preliminary evaluation of MRI texture analysis using the finite difference method and an artificial neural network. AJR Am J Roentgenol, 189(1):117–122.
5. Chen DR, Chang RF, Huang YL (1999) Computer-aided diagnosis applied to US of solid breast nodules by using neural networks. Radiology, 213(2):407–412.
6. Smith TG, Jr., Lange GD, Marks WB (1996) Fractal methods and results in cellular morphology--dimensions, lacunarity and multifractals. J Neurosci Methods, 69(2):123–136.
7. Sanghera B, Banerjee D, Khan A, et al. (2012) Reproducibility of 2D and 3D Fractal Analysis Techniques for the Assessment of Spatial Heterogeneity of Regional Blood Flow in Rectal Cancer. Radiology, 263(3):865–873.
8. Plotnick RE, Gardner RH, Hargrove WW, Prestegaard K, Perlmutter M (1996) Lacunarity analysis: A general technique for the analysis of spatial patterns. Phys Rev E Stat Phys Plasmas Fluids Relat Interdiscip Topics, 53(5):5461–5468.
9. Castellano G, Bonilha L, Li LM, Cendes F (2004) Texture analysis of medical images. Clin Radiol, 59(12):1061–1069.
10. Zhang Y (2012) MRI texture analysis in multiple sclerosis. Int J Biomed Imaging, 2012:762804.
11. Brown RA, Frayne R (2008) A comparison of texture quantification techniques based on the Fourier and S transforms. Med Phys, 35(11):4998–5008.
12. Tuceryan M, Jain AK (1998) Texture analysis. In: Chen CH, Pau LF, Wang PSP (eds) The Handbook of Pattern Recognition and Computer Vision. World Scientific Publishing Co, Singapore, pp 207–248.
13. Arivazhagan S, Ganesan L (2003) Texture segmentation using wavelet transform. Pattern Recog Lett, 24:3197–3203.
14. Mallat SG (1989) A theory of multiresolution signal decomposition: the wavelet representation. IEEE Transactions on Pattern Analysis and Machine Intelligence, 11(7):674–693.
15. Sifuzzaman M, Islam M, Ali M (2009) Application of wavelet transform and its advantages compared to Fourier transform. Journal of Physical Sciences, 13:121–137.
